# Editorial: Dynamic Functional Connectivity in Neuropsychiatric Disorders: Methods and Applications

**DOI:** 10.3389/fnins.2020.00332

**Published:** 2020-04-20

**Authors:** Xiaoya Fu, Feng Liu, Zaixu Cui, Wenbin Guo

**Affiliations:** ^1^Department of Psychiatry, The Second Xiangya Hospital of Central South University, Changsha, China; ^2^National Clinical Research Center on Mental Disorders, Changsha, China; ^3^Department of Radiology, Tianjin Medical University General Hospital, Tianjin, China; ^4^Department of Psychiatry, Perelman School of Medicine, University of Pennsylvania, Philadelphia, PA, United States

**Keywords:** functional magnetic resonance imaging, dynamic functional connectivity, neuropsychiatric disorders, cognitive deficits, regional homogeneity, amplitude of low-frequency fluctuations

Resting-state functional magnetic resonance imaging (RS-fMRI), a non-invasive measurement of spontaneous brain activity, has greatly broadened our understanding of neural substrate underlying neuropsychiatric disorders over the last several decades. Since Biswal et al. discovered synchronized brain activity in different brain areas even without any tasks or stimuli (Biswal, 2012), numerous studies have investigated resting-state coupling (i.e., functional connectivity, FC) between different brain areas in neuropsychiatric disorders (Guo et al., 2015; Zhu et al., 2018).

FC can be defined as a temporal correlation of blood-oxygen-level dependent (BOLD) signal between spatially distributed brain regions (Biswal et al., 1997). Most previous RS-fMRI studies assumed that FC was constant throughout the observation period of task-free experiments (Hutchison et al., 2013). Recently, several studies have demonstrated the feasibility of dynamic methods in characterization of functional brain changes, such as dynamic FC (dFC) investigated by the sliding-window method, which provide novel insights into underlying neural activity (Chang and Glover, 2010; Liu et al., 2017; Duan et al., 2019). However, window size, window stepsize, and window type are open areas of research and important parameters to capture the resting-state FC dynamics. Sliding-window and time-frequency analyses are the two frequently used dynamic functional analyses (Hutchison et al., 2013). Apart from dFC, dynamic amplitude of low frequency fluctuations (dALFF) and dynamic regional homogeneity (dReHo) are also widely used (Deng et al., 2016; Fu et al., 2018). Both static and dynamic functional metrics provide great insight into understanding functional deficits of neuropsychiatric disorders (Biswal, 2012; Hutchison et al., 2013). Therefore, deep and detailed understanding of the method and application of dynamic functional metrics in neuropsychiatric disorders is critical.

This special issue focuses on the recent developments in dynamic functional analyses and their applications in neuropsychiatric disorders. A total of 7 articles were included in this Research Topic.

## PostTraumatic Stress Disorder (PTSD)

Fu et al. applied dReHo and dFC to investigate both local and large-scale functional coupling in patients with PTSD. Results indicated increased dReHo in the left precuneus in patients with PTSD. Also, the left precuneus exhibited increased dFC with the left insula and decreased dFC with the left inferior parietal lobe and right precuneus, suggesting that the left precuneus might be critical for the pathophysiology of PTSD.

## Generalized Anxiety Disorder (GAD)

Brain signal variability (BSV) is a method to measure the temporal variability of standard variation of BOLD signal, which reflects capacity of transition between brain states and processing various external stimuli. Li L. et al. evaluated the changes of BSV in patients with GAD and found that extensive brain regions exhibited decreased BSV in patients with GAD compared to healthy controls (HCs), suggesting that the brain of patients with GAD may be in a less flexible state compared to HCs.

## Early Blind

Dynamic causal modeling (DCM) is an approach to measure causal functional interactions among neuronal populations, i.e., effective connectivity. Li, Wang et al. used spectral DCM to investigate whether early visual deprivation had an impact on the dynamic causal interactions among regions within the default mode network, salience network, and dorsal attention network in patients with early blind. Abnormal patterns of effective connectivity within all these three networks were found in patients with early blind compared to HCs, which might imply the effect of early sensory deprivation on brain plasticity.

## Parkinson's Disease (PD)

Li, Xiong et al. recruited 62 participants with PD and demonstrated that dynamic nodal efficiency measurement, which was calculated from RS-fMRI brain network and sliding-window analysis, could be used to predict the severity level of PD after drug therapy. Hippocampus, post-central gyrus, cingulate gyrus, and orbital gyrus were the contributed regions for the prediction. This study offered an example of using RS-fMRI data to predict the treatment effect in patients with PD.

Another study on PD by Zhang C. et al. used dALFF to explore the feasibility of differentiating patients with PD from HCs. Increased coefficient of variation in the left precuneus was observed in patients with PD. Moreover, coefficient of variation of dALFF in the left precuneus was positively correlated with disease duration in the patients. These findings were likely to provide a new direction for diagnosis of PD.

## Subacute Stroke

Chen et al. explored abnormal dynamic characteristics in patients with subacute stroke. Results of both dALFF and dReHo showed significant intergroup differences of regional brain activity. Fugl-Meyer assessment, an index for evaluating the degree of motor deficit, exhibited a positive correlation with dALFF variability in supplementary motor area (SMA) and a negative correlation with dReHo variability in ipsilesional middle frontal gyrus (MFG). The receiver operating characteristic analysis suggested that dALFF in SMA and dReHo in ipsilesional MFG might be potential markers to distinguish patients with subacute stroke from HCs. Therefore, dALFF and dReHo have the potential for evaluating the motor function in patients with subacute stroke.

## Schizophrenia

Zhang Y. et al. focused dReHo and dynamic fALFF to investigate abnormal dynamic local functional activity in schizophrenia. Results revealed deficits in the sensory and perception functional networks and a positive relationship between dReHo of the thalamus and the severity of symptoms in the patients, which highlighted the importance of the sensorimotor networks in the physiopathology of schizophrenia.

Taken together, all studies in this special issue suggested progress in the methodology and application of dynamic functional properties in neuropsychiatric disorders. These advances would promote better understanding in the temporal evolution of brain functional activity and provide valuable insight into the development of objective neuro-biomarker of neuropsychiatric disorders. Clinicians will be benefit from this topic in regard to theoretical, experimental and clinical questions related to the nature and origins of dFC in neuropsychiatric disorders.

## Author Contributions

WG has contributed to the conception and design of the editorial. XF has not contributed to the conception or design of the editorial, yet contributed considerably to the writing. FL and ZC contributed to the revision of the editorial.

### Conflict of Interest

The authors declare that the research was conducted in the absence of any commercial or financial relationships that could be construed as a potential conflict of interest.
